# Combination of transvaginal ultrasound with cervical cancer screening contributes to early detection of ovarian cancer: Clinical trial

**DOI:** 10.1002/ijgo.70599

**Published:** 2025-11-03

**Authors:** Hiromasa Fujita, Kazuhira Okamoto, Hidenori Kato, Hidemichi Watari, Nobuyoshi Ozawa, Mitsuaki Suzuki

**Affiliations:** ^1^ Cytology Center of Hokkaido Cancer Society Sapporo Hokkaido Japan; ^2^ Department of Obstetrics and Gynecology, Graduate School of Medicine Hokkaido University Sapporo Hokkaido Japan; ^3^ Ozawa Women's General Clinic Senndai Miyagiken Japan; ^4^ Cancer Center, Shin‐Yurigaoka General Hospital Kawasaki Kanagawaken Japan

**Keywords:** clinical stage I, ovarian cancer, screening examination, transvaginal ultrasound, type I, type II

## Abstract

**Objective:**

Early detection of ovarian cancer at stage I is important to improve patients‘ prognosis. The goal of this study was to examine if transvaginal ultrasound (TVU) performed at the same time as cervical cancer screening can facilitate early detection of ovarian cancer.

**Methods:**

From 2014 to 2022, 483 269 women underwent TVU examinations during cervical cancer screening. The criteria for abnormal findings on TVU were ovarian enlargement ≥3 cm in long diameter (≥2 cm in postmenopausal women).

**Results:**

Of the 483 269 women who underwent TVU, 3294 (0.68%) were selected for detailed examination. Of these women, 550 underwent surgery and 80 cases of ovarian cancer were found (positive predictive value: 2.43%). Pathologic review in 76 of these cases showed 70 cases of epithelial ovarian cancer (type I: 54 [77.1%], Type II: 16 [22.9%]) and six cases of non‐epithelial malignant tumors. Clinical staging analysis showed that 81.6% (62 cases) were at stage I. Significantly more type I than type II tumors were detected at stage I (87.3% (*n* = 46) vs. 56.3%, *P* = 0.0068 (*n* = 9)). Notably, 95.7% (22/23) of clear cell carcinoma cases were detected at stage I.

**Discussion:**

The high rate of early detection of type I ovarian cancer might be due to its slow progression. In Asia, where type I is common, the benefits of screening for ovarian cancer are particularly great. However, screening with TVU has generally been considered to have little benefit. The results of this study suggest a need for reassessment of this view.

## 1 INTRODUCTION

In 2022, an estimated 324 603 women worldwide were newly diagnosed with ovarian cancer, and 206 956 women (64%) died from the disease.^1^ In Japan, the incidence of ovarian cancer has increased significantly in recent years, with 13 381 new cases reported in 2019, 1.8 times higher than the 7490 cases in 1999.^2^ Ovarian cancer still has a poor prognosis, partly because a screening system has not been established and many cases are detected at advanced stages of stage II or higher.

Given the poor prognosis associated with ovarian cancer diagnosed at advanced stages, considerable efforts have been directed toward developing effective strategies for earlier detection. Screening methods have included serum CA125 measurement, transvaginal ultrasound (TVU), and combinations of these modalities.^3^, ^4^ The Prostate, Lung, Colorectal and Ovarian (PLCO) Cancer Screening Trial in the United States evaluated annual CA125 testing and TVU in a general population but did not demonstrate a significant reduction in ovarian cancer mortality.^5^ In contrast, the UK Collaborative Trial of Ovarian Cancer Screening (UKCTOCS) investigated a multimodal screening strategy based on longitudinal CA125 trends interpreted using the Risk of Ovarian Cancer Algorithm (ROCA), followed by TVU when indicated. While this approach showed a shift toward earlier‐stage detection, the primary analysis did not reveal a statistically significant mortality reduction.^6^, ^7^ Other studies have assessed the use of CA125 alone for population‐based screening, but its limited sensitivity and specificity, especially for early‐stage disease‐have, constrained its effectiveness. TVU, while capable of detecting adnexal masses, also suffers from low specificity and cannot reliably distinguish between benign and malignant lesion.^8^

As described above, to date, various screening strategies for ovarian cancer have shown limited effectiveness, with no clear reduction in mortality. However, in recent years, remarkable improvements in ultrasound technology have been observed, and an increased detection rate of early‐stage (stage I) cancers is now expected compared to the past. With earlier detection at stage I, prognosis can be significantly improved, as for many other types of cancer. In Hokkaido, which has many medically underserved areas, performance of TVU at the same time as cervical cancer screening was introduced in 2010. The aim of this study is to evaluate whether this approach has contributed to early detection of ovarian cancer.

Screen‐detected epithelial ovarian cancers were classified according to the dualistic model proposed by Kurman and Shih.^9^

Type I tumors (e.g., clear cell, endometrioid, mucinous, and low‐grade serous carcinomas) are generally indolent, genetically stable and frequently associated with *KRAS*, *BRAF*, or *PTEN* mutations. Because they often progress stepwise through precursor lesions, they can be detected at an early stage. However, they tend to be less sensitive to chemotherapy, and once diagnosed at stage II or beyond, the long‐term prognosis is poor.

In contrast, type II tumors, predominantly high‐grade serous carcinomas, are genetically unstable and typically harbor TP53 mutations. As they usually arise de novo without precursor lesions, early‐stage (stage I) detection is challenging.

## 2 MATERIALS AND METHODS

A retrospective study was conducted in 483 269 women who underwent TVU simultaneously with cervical cancer screening at the Hokkaido Cancer Association between 2014 and 2022. TVU was offered as an optional examination for an additional fee of 1000 yen; therefore, 54 092 of the 537 361 women who underwent cervical cancer screening during this period did not receive TVU.

Transvaginal ultrasound was performed with a SONOVISTA FX (Siemens) before 2022, when the instrument was changed to a SONOVISTA GX30. The change from the SONOVISTA FX to the SONOVISTA GX30 in 2022 was part of a routine equipment upgrade. Both devices were operated by the same team of experienced gynecologists using standardized imaging protocols. There were no interruptions in participant recruitment or screening during this transition, and image quality and diagnostic consistency were maintained through internal validation procedures.

The criteria for identifying abnormalities on TVU included ovarian enlargement ≥3 cm in longitudinal diameter (≥2 cm in postmenopausal women), using the International Ovarian Tumor Analysis (IOTA) risk classification system.^10^ In this study, TVU findings were classified into three types based on the IOTA criteria. Type 1 refers to unilocular cysts without solid components, which are generally considered benign. Type 2 includes masses with septations or minimal solid areas but without clear malignant features. Type 3 or higher indicates high‐risk lesions characterized by features suggestive of malignancy, such as papillary projections, solid components, or increased vascularity. Thus, type 3 or higher lesions were considered to be high risk. Even for types 1 and 2, women who were considered candidates for surgery were advised to undergo a comprehensive evaluation at a cancer treatment facility or regional referral center, taking into account factors such as lesion size and patient age. Women who were deemed to require surgical treatment based on the detailed examination were advised to undergo surgery, while those deemed to be at low risk were monitored every 6–12 months.

The classification of no risk of cancer was defined as women with no ovarian cysts or with cysts measuring less than 3 cm in maximum diameter (less than 2 cm in postmenopausal women) on TVU examination. Low risk of cancer was defined as women who were referred for further examination based on TVU findings but had no abnormalities detected on tumor markers such as CA125 or on imaging studies including CT and MRI. Notably, even among women classified as low risk of cancer, surgery was recommended if they had large tumors (≥8–10 cm) or clinical symptoms such as abdominal pain and if a gynecologic oncologist judged the case to be operable.

For cases in which surgery was performed, pathological confirmation and FIGO staging were performed after the initial surgery by board‐certified pathologists and gynecologic oncologists at the respective cancer treatment hospitals or regional core hospitals where the surgeries were conducted. Further, epithelial ovarian cancer was classified into type I and type II and analyzed. The pathological classification and clinical stage of ovarian malignant tumors were based on the 2014 World Health Organization guidelines and FIGO classification, respectively.

In Japanese women, in whom type I ovarian cancers are predominant, we examined the utility of regular TVU by statistically analyzing whether there was a stage difference between type I (*n* = 56) and type II (*n* = 16) ovarian cancers. The statistical analysis was performed using Pearson's χ^2^‐test.

## 3 RESULTS

The ages of the 483 269 women who underwent TVU screening ranged from 18 to 97 years, with a mean age of 56.8 years. The most represented age group was 60–69 years (27.2%), followed by 50–59 years (21.4%) and 40–49 years (20.0%), with women aged 40–69 years accounting for 68.6% of the entire cohort. Regarding race/ethnicity, nearly all participants were Japanese nationals residing in Hokkaido.

Of the 483 269 women who underwent TVU, 3294 were recommended for further examination, yielding a recall rate of 0.68% (3294/483269).

Among these, 2742 were classified as low risk and were followed for 6 to 12 months, during which none progressed to high risk.

**FIGURE 1:**
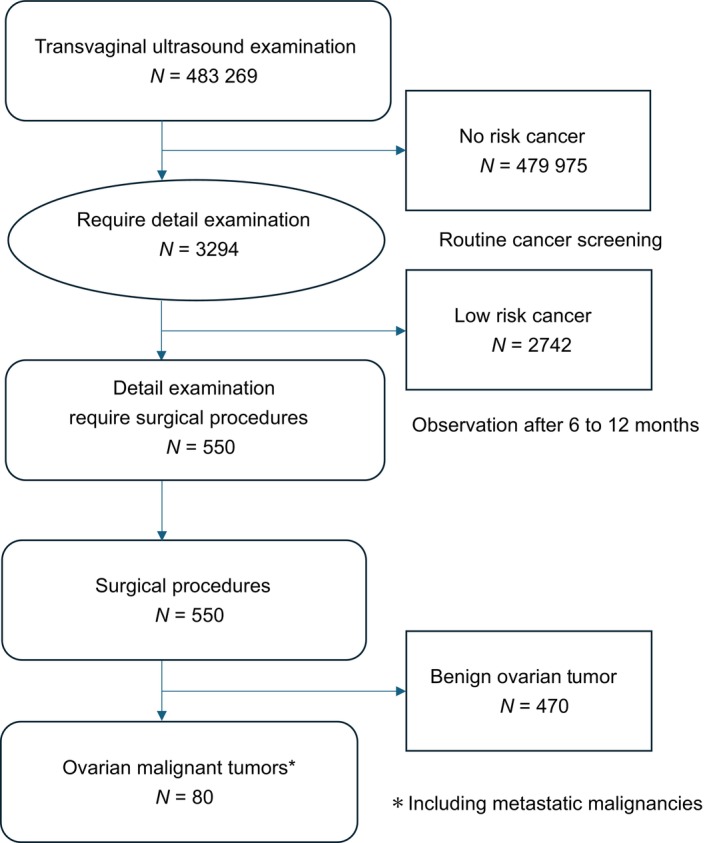
Flow chart for ovarian cancer examination by transvaginal ultrasound. Of the women who underwent TVU, 3294 were recommended for further testing (need rate 0.68%). Of these, 2744 were deemed low risk and were followed for 6–12 months. A total of 550 were deemed high risk or indicated for surgery and underwent surgery. After surgery, pathology revealed that 470 were noncancerous and 80 were ovarian malignant (positive response rate 2.43%). Seventy‐six cases of malignant ovarian tumors were included in the study, excluding metastatic ovarian cancer, and four cases of cancer with unknown histology.

A total of 550 patients who were considered high risk underwent surgery. Postoperative histopathology revealed malignant ovarian tumors in 80 cases and benign ovarian tumors in 470 cases. The positive predictive value (PPV) of the detailed examination was 2.43% (80/3294) (Figure 1). Among the 80 malignant ovarian tumors, two were metastatic ovarian cancers and two were cancers of unknown histology. Excluding these four cases, the histologic types and clinical stages of the 76 cases of malignant ovarian tumors detected are shown in Tables 1 and 2, respectively.

**TABLE 1:** Histopathologic classification of malignant ovarian tumors in the 2014 WHO classification.

Histopathologic classification (WHO)	Numbers (%)	Numbers (%)
Epithelial malignant tumors		
Type I	54 (71.1)	
Low‐grade serous carcinoma		9 (11.8)
Endometrioid carcinoma		9 (11.8)
Mucinous carcinoma		12 (15.8)
Clear cell carcinoma		23 (30.3)
Mixed carcinoma		1 (1.3)
Type II	16 (21.1)	
High‐grade serous carcinoma		15 (19.7)
Undifferentiated carcinoma		1 (1.3)
Non‐epithelial malignant tumors	6 (7.9)	
Adult granulosa cell tumor		5 (6.6)
Immature teratoma		1 (1.3)

*Notes*: Histopathologic classification of malignant ovarian tumors in the 2014 WHO classification. Of the 76 malignant ovarian tumors studied, 70 were epithelial malignant ovarian tumors (54 Type I, 16 Type II) and 6 were non‐epithelial malignant ovarian tumors. The incidence of Type I was 71.1%, that of Type II was 21.1%, and that of non‐epithelial malignant ovarian tumors was 7.9%. The ratio of Type I to Type II was 77.1:22.9. The incidence of clear cell carcinoma of 30.3% was extremely high.

**TABLE 2:** Detection rates of ovarian malignant tumor by clinical stage and by clinical stage for different types (FIGO).

Clinical stage (FIGO)	All ovarian malignant tumors (*n* = 76)	Epithelial type		Non‐epithelial type
		Type I (*n* = 54)	Type II (*n* = 16)	Other types (*n* = 6)
Stage I	62 (81.6%)	47 (87.0%)	9 (56.3%)	6 (100%)
Stage II	2 (2.6%)	1 (1.9%)	1 (6.3%)	0
Stage III	10 (13.2%)	6 (11.0%)	4 (25.0%)	0
Stage IV	2 (2.6%)	0.0%	2 (12.5%)	0

*Notes*: Pearson's χ^2^‐test: *P* = 0.0068. The 76 ovarian malignant tumors were classified into four stages (FIGO classification): 62 were stage I, 2 were stage II, 10 were stage III, and 2 were stage IV, with stage I accounting for 81.6% of the total. Of the 70 epithelial carcinomas, 54 were type I, 47 (87.0%) were stage I, and 7 (12.9%) were stage II‐IV. Of the 16 type II carcinomas, 9 (56.3%) were stage I, and 7 (43.8%) were stage II‐IV. There was a significant difference between the two groups (Pearson's chi‐square test, *P* = 0.0068). Furthermore, 95.7% (22/23) of type I clear cell carcinomas were stage I, and all 6 non‐epithelial ovarian tumors were stage I.

Of the 76 malignant ovarian tumors, 70 were epithelial ovarian cancers (type I: 54, type II: 16) and six were non‐epithelial ovarian malignant tumors. The 70 epithelial cancers were low‐grade serous carcinoma (*n* = 9), clear cell carcinoma (*n* = 23), endometrioid carcinoma (*n* = 9), mucinous carcinoma (*n* = 12), mixed carcinoma (*n* = 1), high‐grade serous carcinoma (*n* = 15), and undifferentiated carcinoma (*n* = 1). The six non‐epithelial tumors were adult granulosa cell tumor (*n* = 5) and immature teratoma (*n* = 1) (Table 1).

In clinical staging, the 76 malignant ovarian tumors were classified as stage I (*n* = 62), II (*n* = 2), III (*n* = 10), and IV (*n* = 2), with stage I cases accounting for 81.3% of all cases. In grouping by type, 46 type I cases (87.0%) were stage I and seven (12.8%) were stage II–IV, while nine type II cases (56.3%) were stage I and seven (43.8%) were stage II–IV, with a significant difference between the types (*P* = 0.0068, Pearson *χ* ^2^‐test) (Table 2). Among type I tumors, clear cell carcinoma had a particularly high early‐stage detection rate, with 95.7% (22/23) classified as stage I. All six cases of non‐epithelial ovarian tumors were also stage I.

## 4 DISCUSSION

In large trials such as PLCO and UKCTOCS (5, 6), the effectiveness of TVU for ovarian cancer screening has been limited. However, in a recent supplementary analysis of the UKCTOCS, Menon et al.^11^ examined patients with high‐grade serous carcinoma and found that the multimodal screening group—combining TVU and serum CA125 measurements—had a significantly higher survival rate compared to the non‐screening group. This result has led to re‐evaluation of the effectiveness of ovarian cancer screening. The new finding that screening tests using TVU can improve the prognosis of ovarian cancer is thought to be largely due to recent advances in treatment, as well as improvements in the performance of ultrasound equipment.^12^, ^13^ The PLCO and UKCTOCS trials were conducted from 1993 to 2005, and at that time the 5‐year survival rate for ovarian cancer was 29%–40% in the USA (2001–2003)^14^ and 36% in Europe (1995–2002).^15^ In contrast, the current 5‐year survival rate exceeds 50% due to the use of new anticancer drugs and molecularly targeted drugs.^16^, ^17^ The 5‐year survival rate for stage I (localized) cancer is now over 90% and the 10‐year survival rate has reached 85%.^18^, ^19^, ^20^ In the current study, the detection rate was 81.3% for **s**tage I cancer and 87.0% for type I ovarian cancer only. We were unable to analyze prognosis, but it is likely that the high detection rate for stage I cancer will lead to improved prognosis for ovarian cancer.

The high detection rate of stage I cancer might be due to the improved performance of ultrasound equipment and the fact that the practitioners were gynecologists. However, the main reason is likely to be that most of the detected cancers were type I ovarian cancers. Type I ovarian cancer progresses relatively slowly, so regular screening increases the chance of early detection. The types of ovarian cancer that occur vary by ethnicity and region, with type II being more common in Europe and the USA and type I being more common in Asia, including Japan.^21^, ^22^, ^23^ In Japan, type I cases account for 65% of ovarian cancers,^24^ and the incidence of clear cell carcinoma is high among type I ovarian cancers.^25^ Thus, detection at stage I is important to improve the prognosis of clear cell carcinoma. In this study, 23 cases of clear cell carcinoma were found, of which 22 (95.7%) were stage I. This is likely to contribute greatly to improving the prognosis of this tumor.^26^, ^27^

The results of ovarian cancer screening in the present study, which targeted residents of Hokkaido, are likely applicable to the broader Japanese population, given the similarity in race/ethnicity. Further, the findings might be generalizable to other Asian populations where type I ovarian cancer is also relatively prevalent. In contrast, in Europe and the USA, where type II ovarian cancers are more common, the impact of screening on increasing the detection of stage I ovarian cancer might be limited.

In this study, abnormal findings on TVU were assessed using the IOTA model. Compared to the Risk of Malignancy Index (RMI), the IOTA models—particularly the ADNEX model—offer superior sensitivity and specificity for distinguishing between benign and malignant ovarian tumors and allow for more detailed risk assessment. The ADNEX model estimates not only the overall risk of malignancy but also subtype‐specific risks, such as borderline tumors or early‐ and advanced‐stage cancers. Recent international guidelines recommend IOTA models over RMI. Considering their compatibility with cervical cancer screening and ease of interpretation by gynecologists performing TVU, the IOTA criteria were adopted. Also, this study was designed to promote the detection of ovarian cancer by adding TVU to the cytology‐based cervical cancer screening program (a population‐based screening program in Japan). Blood sampling was not conducted, and measurements such as CA125 were not performed.

Cervical cancer screening in Japan has a history of 70 years, and quality control has been established.^28^ It is not difficult to include TVU in the cervical cancer screening system, and there is no significant increase in cost if TVU is performed at the same time as screening. Addition of TVU as an option is estimated to cost an extra 1000 yen. For residents of sparsely populated areas such as those in Hokkaido, regular cervical cancer screening every 2 years provides an important opportunity to detect both cervical cancer and ovarian and endometrial cancers.

In cancer screening tests, the problem of false‐positive results must also be considered. Specifically, we noted that false positives in ovarian cancer screening can lead to unnecessary diagnostic procedures or even surgery, which may impose psychological stress, physical harm, and additional medical costs. Therefore, minimizing false positives is an important consideration when evaluating the overall effectiveness and feasibility of a screening program.

Unlike cervical or breast cancer, ovarian cancer usually cannot be diagnosed preoperatively by biopsy, making it difficult to obtain a definitive diagnosis before surgery. Thus, the indication for surgery requires particular caution. In our study, the referral rate for further examination was as low as 0.68%, which is lower than that reported in screening programs for other cancers such as cervical or breast cancer. From this perspective, the relatively low referral rate observed in our program might be considered reassuring. In ovarian cancer screening using TVU in this study, the PPV was 2.46%. For comparison, the PPVs for organized cancer screening in Japan are 1.20% for cervical cancer, 2.8% for colorectal cancer, and 4.15% for breast cancer.^29^ Thus, the PPV in this study is comparable to other organized cancer screenings and is considered acceptable.

The results of this study show that incorporating TVU into cervical cancer screening in Hokkaido enabled the detection of a substantial number of early‐stage ovarian cancer cases. This method is particularly useful for early detection of type I ovarian cancer, such as clear cell carcinoma. TVU has been considered to be of little value in screening for ovarian cancer, but the results of this study suggest that it is time to reconsider the utility of this method.

It would also be useful to evaluate the effectiveness of TVU in ovarian cancer detection using the number needed to treat (NNT) as an indicator. However, because this study did not include a control group, it was unfortunately not possible to calculate or present the NNT. This represents one of the limitations of our study.

## 5 STRENGTHS AND LIMITATIONS

The strengths of this study include the integration of TVU into the conventional cervical cancer screening system in Japan, thereby enabling simultaneous ovarian cancer screening. A total of 483 269 women underwent screening, and 80 cases of ovarian cancer were detected, more than 80% of which were stage I disease. These findings highlight the potential utility of TVU for the early detection of ovarian cancer. However, several limitations should be acknowledged. First, because this was not a randomized controlled study, there was no control group, and parameters such as the number needed to treat could not be evaluated. Second, the follow‐up period was relatively short, which precluded assessment of the long‐term prognosis of patients with ovarian cancer. Despite these limitations, our study provides important evidence supporting the feasibility and potential clinical value of incorporating TVU into population‐based cancer screening programs in Japan.

## AUTHOR CONTRIBUTIONS

Hiromasa Fujita, Nobuyoshi Ozawa, and Mitsuaki Suzuki were responsible for the conception and design of the study. Hiromasa Fujita, Kazuhira Okamoto, and Hidenori Kato were responsible for data collection and validation. Hiromasa Fujita performed data and statistical analysis under the guidance of Hidemichi Watari, Nobuyoshi Ozawa, and Mitsuaki Suzuki. All authors interpreted the data. Hiromasa Fujita prepared the manuscript. All authors thoroughly reviewed the manuscript, approved the final version, and take responsibility for its integrity. Mitsuaki Suzuki oversaw the study's implementation. All authors had access to all data in the study and agreed to be responsible for the decision to submit.

## CONFLICT OF INTEREST STATEMENT

The authors have no conflicts of interest to declare.

## ETHICS STATEMENT

This study was approved by the Ethics Committee of the Hokkaido Cancer Society.

## CONSENT

Prior to participating in this study, written consent was obtained for use of data. To safeguard the privacy of the subjects, no personally identifiable information was utilized.

## Data Availability

Research data are not shared.
